# Autoimmune Cytopenias In Common Variable Immunodeficiency

**DOI:** 10.3389/fimmu.2012.00189

**Published:** 2012-07-24

**Authors:** Jenna C. Podjasek, Roshini S. Abraham

**Affiliations:** ^1^Division of Allergic Diseases, Department of Medicine, Mayo Clinic, Rochester, MN, USA; ^2^Cellular and Molecular Immunology Laboratory, Division of Clinical Biochemistry and Immunology, Department of Laboratory Medicine and Pathology, Mayo Clinic, Rochester, MN, USA

**Keywords:** common variable immunodeficiency (CVID), autoimmune cytopenias, immune thrombocytopenia, autoimmune hemolytic anemia, autoimmune lymphoproliferative syndrome, Evans syndrome

## Abstract

Common variable immunodeficiency (CVID) is a humoral immunodeficiency whose primary diagnostic features include hypogammaglobulinemia involving two or more immunoglobulin isotypes and impaired functional antibody responses in the majority of patients. While increased susceptibility to respiratory and other infections is a common thread that binds a large cross-section of CVID patients, the presence of autoimmune complications in this immunologically and clinically heterogeneous disorder is recognized in up to two-thirds of patients. Among the autoimmune manifestations reported in CVID (20–50%; Chapel et al., 2008; Cunningham-Rundles, 2008), autoimmune cytopenias are by far the most common occurring variably in 4–20% (Michel et al., 2004; Chapel et al., 2008) of these patients who have some form of autoimmunity. Association of autoimmune cytopenias with granulomatous disease and splenomegaly has been reported. The spectrum of autoimmune cytopenias includes thrombocytopenia, anemia, and neutropenia. While it may seem paradoxical “*prima facie*” that autoimmunity is present in patients with primary immune deficiencies, in reality, it could be considered two sides of the same coin, each reflecting a different but inter-connected facet of immune dysregulation. The expansion of CD21 low B cells in CVID patients with autoimmune cytopenias and other autoimmune features has also been previously reported. It has been demonstrated that this unique subset of B cells is enriched for autoreactive germline antibodies. Further, a correlation has been observed between various B cell subsets, such as class-switched memory B cells and plasmablasts, and autoimmunity in CVID. This review attempts to explore the most recent concepts and highlights, along with treatment of autoimmune hematological manifestations of CVID.

## INTRODUCTION

Common variable immunodeficiency (CVID) is a highly heterogeneous immunodeficiency with varying complexity. The key diagnostic elements include low IgG (2 SD below mean of age) along with low IgA and/or IgM (Park et al., 2008; Resnick et al., 2011). CVID is considered the most commonly encountered and clinically relevant primary immunodeficiency in adults (Chapel et al., 2008; Park et al., 2008) and though the majority of patients are diagnosed between the age of 20 and 40 years, at least another 20% are diagnosed during childhood (>2 years) or adolescence (Cunningham-Rundles, 2010).

While recurrent sinopulmonary infections are one of the hallmarks of this disease, gastrointestinal, viral, and systemic bacterial infections have also been reported (Park et al., 2008; Resnick et al., 2011). Besides infections, CVID is associated with a variety of non-infectious complications including pulmonary disease, autoimmunity, granulomatous disease, gastrointestinal disease, and malignancy (Chapel et al., 2008; Resnick et al., 2011).

The clinical heterogeneity and complexity of CVID has led to renewed efforts over the past decade to identify causal genetic defects as well as correlate the “immuno-phenotype” with clinical phenotype (Warnatz et al., 2002; Piqueras et al., 2003; Wehr et al., 2008; Eibel et al., 2010). In the last 10 years, monogenic defects associated with antibody deficiency have been described in a small subset of CVID patients or patients with hypogammaglobulinemia, or single or few families with a history of consanguinity. These genetic defects include disease-causing mutations or polymorphisms in the *TNFRSF13B* (TACI), *CD19*, *ICOS*, *TNFRSF13C* (*BAFF-R*), *CD81*, *CD20*, *MSH5*, and *CD21* genes (Grimbacher et al., 2003; Salzer et al., 2004, 2005, 2009; Castigli et al., 2005, 2007; Warnatz et al., 2005; van Zelm et al., 2006, 2010; Kanegane et al., 2007; Pan-Hammarstrom et al., 2007; Schaffer et al., 2007; Sekine et al., 2007; Zhang et al., 2007; Kuijpers et al., 2010; Frank, 2012; Thiel et al., 2012). However, single-gene defects were identified in only a relatively small subset of CVID patients raising the possibility that the majority (>75%) of CVID patients have oligogenic or polygenic defects. This was recently substantiated by a genome-wide association study of 363 CVID patients, which revealed that copy number variations (CNV), including gene duplications and/or deletions were present and this analysis led to the identification of several “novel” genes, which may play an important role in the immune response, and genetic variations therein could lead to a disease phenotype associated with CVID (Orange et al., 2011).

Paradoxical as it may seem, autoimmune manifestations are not uncommon in patients with primary immunodeficiencies (PIDDs) and at least 25% of all PIDDs described in the 2011 IUIS classification may have some form of autoimmune phenomenon (Bussone and Mouthon, 2009; Notarangelo, 2009; Al-Herz et al., 2011). The autoimmunity observed in PIDDs may be related either to a direct or indirect genetic effect, and includes defects in genes that regulate immunological self-tolerance as well as genetic variations that alter immune regulation. Not surprisingly, therefore, autoimmune features are identified relatively frequently in CVID patients (Brandt and Gershwin, 2006; Knight and Cunningham-Rundles, 2006; Cunningham-Rundles, 2008).

## AUTOIMMUNITY IN CVID

Autoimmune hematological abnormalities, specifically cytopenias, are the most common of all autoimmune manifestations in CVID and may present as thrombocytopenia, anemia or neutropenia. In the longitudinal study mentioned above, immune thrombocytopenia (ITP) was reported in 14% of patients, while autoimmune hemolytic anemia (AIHA) and neutropenia was less common with only 7 and <1%, respectively, of the cohort affected (Resnick et al., 2011). It should also be kept in mind that autoimmune cytopenias may in fact be the presenting symptom for a small subset of CVID patients, especially in children, where Evans syndrome (ES) has been reported to precede the clinical and immunological phenotype of CVID (Savasan et al., 2007). Other autoimmune presentations reported in CVID include rheumatoid arthritis, anti-IgA antibodies, vitiligo, and alopecia (Horn et al., 2007; Park et al., 2008; Resnick et al., 2011). A very recent longitudinal study assessing clinical complications that cause morbidity and mortality in CVID patients identified autoimmune complications in 29% of a cohort of 473 patients studied over 4 decades (Resnick et al., 2011). Interestingly, in the same study, the presence of autoimmunity was not associated with an increase in mortality.

## IMMUNOLOGICAL AND PHENOTYPIC MANIFESTATIONS OF AUTOIMMUNE CYTOPENIAS IN CVID

As alluded to previously, several clinical and immunological classifications have been posited in an attempt to stratify and may be even simplify the complex and heterogeneous phenotypes seen in CVID (Warnatz et al., 2002; Piqueras et al., 2003; Chapel et al., 2008; Wehr et al., 2008). The relatively more recent EUROclass study attempted to cohesively link the earlier Freiburg and Paris classifications by correlating B cell subset immunophenotypes with clinical presentation specifically providing correlation for autoimmunity, granulomatous disease, and splenomegaly (Warnatz et al., 2002; Piqueras et al., 2003; Wehr et al., 2008). Of particular relevance was the correlation of an expansion of CD21^low/dim^ B cells with splenomegaly (Wehr et al., 2008). The CD21^low/dim^ B cells have been previously reported to be a subset of anergic B cells with defective signaling that has the capacity to home to sites of inflammation (Rakhmanov et al., 2009, 2010; Foerster et al., 2010; Charles et al., 2011). Additionally, correlations were identified between an expansion of transitional B cells with lymphadenopathy and autoimmune cytopenias with reduced plasmablasts – pre-terminally differentiated plasma cells (Wehr et al., 2008).

Data from Sanchez-Ramon et al. (2008) and Vodjgani et al. (2007) provide independent substantiation of the association between low class-switched memory B cells and clinical features of autoimmunity and splenomegaly in CVID patients reported by the EUROclass and other classification studies (Warnatz et al., 2002; Piqueras et al., 2003; Wehr et al., 2008).

Martinez-Gamboa et al. (2009) showed that there was a numerical decrease in memory B cell numbers in ITP patients who underwent splenectomy and alluded to a potential role for the spleen in maintaining memory B cell homeostasis. However, a different study suggests that the age at which splenectomy is performed is more relevant to maintenance of marginal zone (memory) B cells numbers than consideration of splenectomy in isolation, regardless of age at which the procedure is done (Wasserstrom et al., 2008).

Besides the correlation of B cell subsets, specifically switched memory B cells, with autoimmunity, there is evidence from multiple human and mouse models on the significance and importance of regulatory T cells expressing FOXP3 in suppressing or controlling autoimmunity (Buckner, 2010; Long and Buckner, 2011). It has been shown in at least a subset of CVID patients, particularly those with autoimmune features, that there is a substantial decrease in relative frequency (%) but not absolute quantitation of FOXP3+ Tregs raising the possibility of abnormal immune regulation in these patients (Arumugakani et al., 2010), though the mechanism of immune dysregulation in this context may extend beyond numerical changes to possible functional alterations as well (Jang et al., 2011; Long and Buckner, 2011).

Another recent study demonstrated B cell receptor recombination bias in a subset of CVID patients and postulated that this may predispose to decreased secondary recombination with subsequent defective central tolerance leading ultimately to the escape of autoreactive clones (Romberg et al., 2011). Further, a biomarker (soluble BAFF/BLys) produced by monocytes and dendritic cells (DCs), which is a critical B cell survival and proliferation factor, and known to be abnormally increased in contexts of autoimmunity, especially in rheumatologic diseases (Becker-Merok et al., 2006) was also been shown to be elevated in CVID patients but there was no demonstrable correlation with the incidence of autoimmunity (Knight et al., 2007).

## CVID: OVERLAP WITH AUTOIMMUNE LYMPHOPROLIFERATIVE SYNDROME AND EVANS SYNDROME

Published data have demonstrated a clear immunologic and clinical overlap between CVID, ES, and autoimmune lymphoproliferative syndrome (ALPS). ES is characterized by the presence of autoimmune cytopenias in two or more hematopoietic lineages. A small study evaluating 12 pediatric patients with ES determined that half (6/12) also had elevated αβ TCR+ DNT T cells (CD3+CD4–8–) and defective Fas apoptosis characteristic of ALPS patients (Teachey et al., 2005). A subsequent larger study of 45 patients with ES substantiated the earlier finding by demonstrating diagnostic criteria for ALPS in 21/45 patients (Seif et al., 2010).

The correlation between ES, ALPS, and CVID was made in a different study, which though limited in sample size (*n* = 7), showed development of hypogammaglobulinemia, as seen in CVID in 5/7 patients with ES. These patients also had increased Fas expression (Savasan et al., 2007). A larger cohort study of 68 patients with ES showed that only a relatively small proportion, 4/68 had CVID (Michel et al., 2009).

In a separate study of ALPS patients (*n* = 66), an equally small number, 5/66 had hypogammaglobulinemia, suggesting a potential phenotypic overlap with CVID. The majority of the ALPS patients in this study had reduced class-switched memory B cells, similar to what has been reported in two-third or greater of CVID patients (Rensing-Ehl et al., 2010).

## MECHANISMS OF DEVELOPMENT OF AUTOREACTIVITY

The development of self-reactive B cells is regulated both centrally (bone marrow) and peripherally through at least two independent check-points. It has been suggested that there may be a failure of both central and peripheral tolerance mechanisms in CVID due to immune dysregulation resulting in a flawed negative selection process. Logically, this would suggest that there would be an increased selection of autoreactive B cells prior to affinity maturation (somatic hypermutation) or memory B cell/plasma cell commitment in the secondary lymphoid organs (Haymore et al., 2008). This is a topic that is discussed in depth elsewhere in this journal series, and therefore, not addressed herein.

## Diagnosis and Treatment

### DIAGNOSIS

**FIGURE 1:**
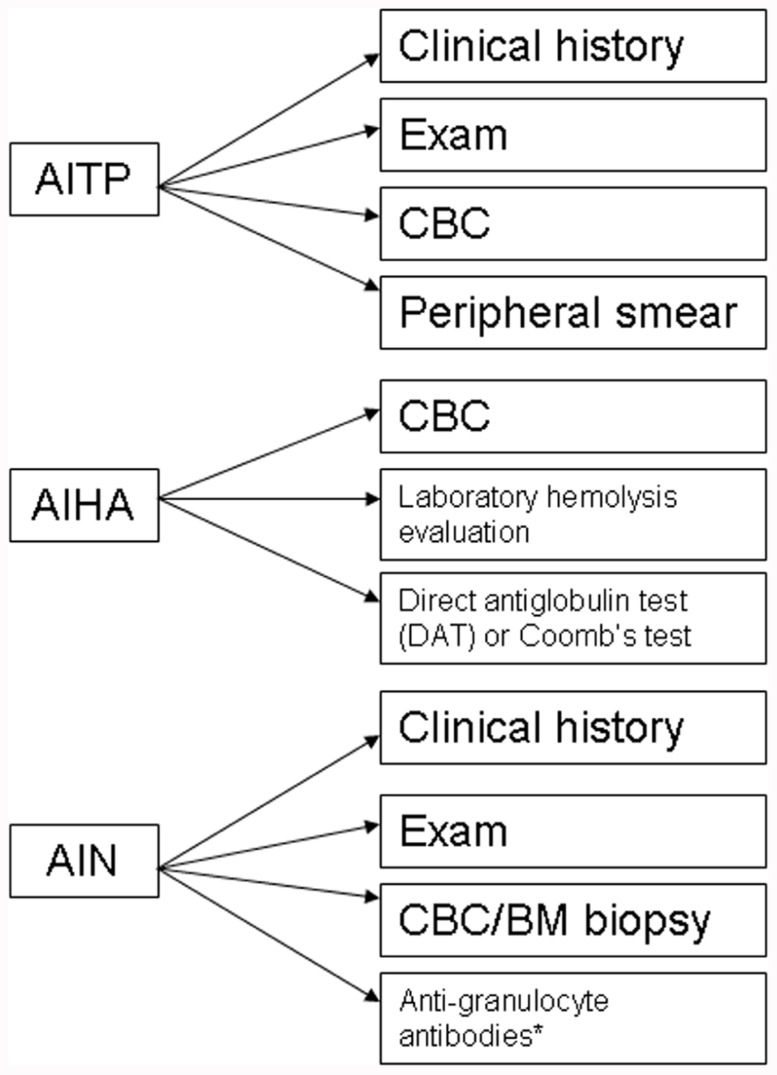
**Diagnostic algorithm for autoimmune cytopenias in CVID**. AITP, autoimmune thrombocytopenia purpura; AIHA, autoimmune hemolytic anemia; AIN, autoneutropenia; IVIG, intravenous immunoglobulin;. *non-exclusionary.

The evaluation of CVID patients for autoimmune cytopenias should include appropriate diagnostic work-up (**Figure 1**), however, in the case of ITP this may primarily be a diagnosis of exclusion. A presumptive diagnosis of ITP can be arrived at by ruling out alternative pathological mechanisms through clinical history, physical review, complete blood count (CBC) analysis, and peripheral blood smears (Provan et al., 2010). Confirmation of the diagnosis is usually determined by response to appropriate treatment. As per the previous discussion that autoimmune cytopenias may precede a diagnosis of CVID, it would be reasonable to evaluate both pediatric and adult patients for immunoglobulin levels on diagnosing ITP to rule out a possible CVID or selective IgA deficiency (Provan et al., 2010). Additionally, follow-up may be required with periodic evaluation and correlation with clinical history to document evolution of the disease process.

Likewise, the diagnosis of AIHA mandates evidence of hemolysis along with detection of an autoantibody. There are a number of laboratory markers for establishing hemolysis, including a CBC with peripheral smear, increased indirect bilirubin, increased lactate dehydrogenase (LDH), and decreased haptoglobin. Autoantibodies can be detected by a direct antiglobulin test (DAT) or Coomb’s test (Gehrs and Friedberg, 2002).

The diagnosis of autoimmune neutropenia (AIN) is similar to ITP in that it is a diagnosis of exclusion. In some cases, detection of anti-granulocyte antibodies may be useful but the lack of detectable autoantibodies does not exclude a diagnosis of AIN (Bope and Kellerman, 2012). Most cases of AIN are associated with normal marrow reserve and pathogenesis is related to antibody-mediated destruction and in some cases, sequestration. The diagnosis can include a bone marrow biopsy, which would reveal a hypercellular marrow and usually a late maturational arrest, though in some cases, an early arrest can also be seen. AIN may be associated with ITP and/or AIHA in CVID patients. Besides, the possible presence of anti-neutrophil antibodies, circulating immune complexes may also be present in a subset of patients with AIN (Dinauer and Coates, 2009).

### TREATMENT

**FIGURE 2:**
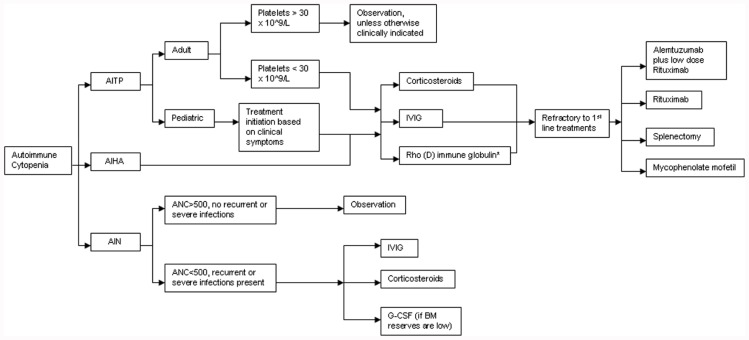
**Treatment algorithm for autoimmune cytopenias in CVID**. AITP, autoimmune thrombocytopenia; AIHA, autoimmune hemolytic anemia; AIN, autoneutropenia; IVIG, intravenous immunoglobulin; ANC, absolute neutrophil count; G-CSF, granulocyte colony stimulating factor. *Rh+, non-splenectomized individuals only.

A treatment algorithm for autoimmune cytopenias in CVID is provided in **Figure 2**. The American Society of Hematology has provided guidelines for the treatment of patients with ITP and these include initiation of treatment in adult patients if the platelets are below 30 × 10^9^/L. However, in pediatric patients, the current guidelines state that treatment is based on clinical symptoms associated with thrombocytopenia regardless of the platelet counts (Neunert et al., 2011). Pediatric patients are far more likely to experience spontaneous remissions. The treatment of choice as first-line therapy for ITP is the use of steroids at 1 mg/kg for a duration of at least three weeks with subsequent dose reduction and eventual withdrawal. Alternative therapeutic options could include a single dose of intravenous immunoglobulin (IVIG) at 1 g/kg. Further use of IVIG is dependent on clinical response to the initial dose. A combination of the above two therapies may be utilized if a rapid response is required. Rho(D) immune globulin is an option for Rh-positive individuals who have not undergone a splenectomy and are unable to tolerate steroid treatment (Neunert et al., 2011). Splenectomy is recommended as a therapeutic option only for those patients that fail corticosteroid therapy. CVID patients undergoing splenectomy or receiving immunosuppressive medication may be at increased risk for infection given their intrinsic immunological defects.

While AIHA is treated much like ITP, it may be more challenging to manage, particularly in patients with ES (Cunningham-Rundles, 2002; Wang and Cunningham-Rundles, 2005). For refractory cases of ITP, AIHA, or both, Rituximab, a chimeric monoclonal anti-CD20 B cell-depleting agent, has been effectively used. In a modest-size cohort of CVID patients (*n* = 33) with refractory autoimmune cytopenias (failure of at least 2–6 treatments prior to initiation of Rituximab), the initial response rate was remarkably high at 84% (Gobert et al., 2011). Severe infection was an unfortunate consequence in almost a quarter of these patients (8/33) over a mean follow-up period of 39 months. Of note, half the patients (4/8) were not on replacement immunoglobulin therapy at the time of infectious diagnosis. An earlier study reports similar rates of infection in patients with ITP who received standard treatment (Michel et al., 2004).

The treatment of AIN is primarily dictated by the severity of neutropenia-associated clinical symptoms and the underlying disease context. Treatment with high-dose IVIG or steroids may be used if there is very profound neutropenia (ANC < 500/mm^3^) in conjunction with recurrent or fulminant infections. G-CSF therapy is only of value if bone marrow reserves are depleted. Splenectomy has little value in reversing neutropenia, especially if it is isolated, since the effect is transient, and can ultimately increase overall infection risk (Dinauer and Coates, 2009).

A separate study of 19 adult patients with steroid-refractory autoimmune cytopenias, reported a 100% initial response rate to a combination of low-dose Rituximab and Alemtuzumab (anti-CD52 humanized monoclonal antibody). Infection occurred in 6/19 patients after a median period of 70 weeks (Gomez-Almaguer et al., 2010). Other reports have documented an initial response rate of 78–92% for refractory autoimmune cytopenias treated with mycophenolate mofetil with no significant adverse events reported (Kotb et al., 2005; Rao et al., 2005). Thus, the approach to treating autoimmune cytopenias in CVID is not dissimilar to the treatment of immune competent patients (Wang and Cunningham-Rundles, 2005).

## SUMMARY

This minireview, which is limited in scope, provides an encapsulated discussion on the incidence and presentation of autoimmunity in CVID, specifically autoimmune cytopenias, their overlap with other clinical entities, some notable immunological hallmarks, laboratory diagnosis and an overview of standard and new therapies. As mentioned in the text, a more exhaustive treatment of autoimmunity in CVID, focusing on mechanistic aspects, is provided elsewhere.

## Conflict of Interest Statement

The authors declare that the research was conducted in the absence of any commercial or financial relationships that could be construed as a potential conflict of interest.
